# The impact of abdominal and laparoscopic hysterectomies on women’s sexuality and psychological condition

**DOI:** 10.4274/tjod.71245

**Published:** 2016-12-15

**Authors:** Meryem Kürek Eken, Gülşah İlhan, Osman Temizkan, Evrim Erbek Çelik, Dilşad Herkiloğlu, Ateş Karateke

**Affiliations:** 1 Adnan Menderes University Faculty of Medicine, Department Obstetrics and Gynecology, Aydın, Turkey; 2 Süleymaniye Training and Research Hospital, Clinic of Obstetrics and Gynecology, İstanbul, Turkey; 3 Şişli Hamidiye Etfal Training and Research Hospital, Clinic of Obstetrics and Gynecology, İstanbul, Turkey; 4 Zeynep Kamil Training and Research Hospital, Clinic of Psychiatry, İstanbul, Turkey; 5 Zeynep Kamil Training and Research Hospital, Clinic of Obstetrics and Gynecology, İstanbul, Turkey

**Keywords:** total abdominal hysterectomy, Total laparoscopic hysterectomy, sexual function, self-esteem, Quality of life

## Abstract

**Objective:**

To investigate whether there were any differences in the quality of life, sexual function, and self-esteem of patients who underwent total laparoscopic hysterectomy (TLH) (n=42) and total abdominal hysterectomy (TAH) (n=42).

**Materials and Methods:**

All premenopausal patients who underwent TLH or TAH because of benign uterine disorders were enrolled. The sexual function and quality of life status were assessed preoperatively and 6 months postoperatively using three standardized validated questionnaires: the Arizona Sexual Experiences Scale (ASEX), the Symptom Checklist-90-Revised (SCL-90-R), and the Rosenberg Self-Esteem Scale (RSES).

**Results:**

Preoperative ASEX, SCL-90-R and RSES scores were not different among the hysterectomy subgroups. The postoperative SCL-90-R scores were also not different among the hysterectomy subgroups. The postoperative RSES scores were significantly lower (p<0.05) than the preoperative scores for all procedures (indicating improved self-esteem) but did not differ among the groups. The postoperative ASEX scores were significantly decreased (p<0.01) as compared with the preoperative scores (indicating improved sexual function). When the average score of each item of the ASEX score was compared in both groups, significant differences were observed in sexual drive and arousal in the laparoscopy group (p<0.01).

**Conclusion:**

Women undergoing TLH for benign uterine disease may have better outcomes related to certain sexual function parameters than women undergoing TAH.

## INTRODUCTION

Hysterectomy is one of the most common gynecologic operations performed in developed countries and is usually performed for benign disorders^(1,2)^.

Sexual function and the quality of life have been the focus of many recent studies. Investigation of the effects of hysterectomy performed for benign indications on sexual function and postoperative quality of life have led to varied results. Hysterectomy may cause sexual dysfunction in the postoperative period^(3,4)^. On the other hand, abnormal uterine bleeding, endometriosis, and adnexal or uterine pathologies can lead to sexual problems, and pain reduce quality of life^(5)^. The rapid progress made in laparoscopic surgery over the past 20 years has been a crucial development in gynecologic surgery and plays a role in the determination of treatment^(6)^. The most significant benefits of laparoscopic surgery include decreased blood loss, lower risk of surgical site infections, shorter hospital stays, and rapid return to normal daily activities in laparoscopy compared with laparotomy^(7)^. However, studies investigating postoperative psychological effects and effects on sexual function are limited.

In the present study, we conducted a prospective cohort trial investigating the advantages and potential drawbacks regarding the sexual function of patients who underwent laparoscopic and abdominal hysterectomy. Our goal was to investigate whether there were any differences in the quality of sexual function postoperatively.

## MATERIALS AND METHODS

The present prospective cohort study was conducted in Zeynep Kamil Training and Research Hospital, which is one of the largest tertiary teaching hospitals in Turkey. All patients provided informed consent. Approval from the local ethics committee was acquired. Preoperatively, all patients underwent gynecologic examination, transvaginal ultrasounds, medical histories were obtained, and routine laboratory tests were performed.

Patients for whom hysterectomy without concurrent unilateral or bilateral adnexectomy was indicated for a benign gynecologic condition, who had a stable heterosexual relationship for at least 1 year, and who had no psychiatric disorders were included in the study. The exclusion criteria were suspicion of malignancy, a previous lower midline incision, the need for simultaneous interventions such as prolapse repair, the need for intraoperatively diagnosed adnexal pathology requiring subsequent unilateral or bilateral oophorectomy, having preoperative or postoperative hormone-therapy, and an inability to communicate in Turkish. In addition, patients with psychiatric disorders, vaginismus, lack of orgasm, a history of endometriosis, partners with sexual dysfunction, those who refused the interview, lost to follow-up, loss of sexual interest, psychological and physiological problems with partner’s relationship, and in postmenopausal period were excluded from the study. The patients and their physician had decided to the route of hysterectomy. The patients were included in the study if they were scheduled for abdominal or laparoscopic hysterectomy. Patients were divided into two groups according to the surgical treatment: the total abdominal hysterectomy (TAH) group and the total laparoscopic hysterectomy (TLH) group. The demographic characteristics (age, marital status, education, and occupation) were recorded. The sexual function and quality of life were assessed 1 day preoperatively on admission to our hospital. Both of the groups’ patients were contacted 6 months postoperatively and interviewed again. All questionnaires were coded with an identifying number, and women could not view their previous answers. We confirmed that all of the patients had had sexual intercourse again six months after surgery. All data were recorded and analyzed by another researcher who was blinded to the group assignments.

### Operating procedures

All patients underwent general anesthesia and received preoperative antibiotic prophylaxis as well as anticoagulants during immobilization. Laparoscopic hysterectomies were all intentionally TLHs^(8)^ and TAH was performed using the standard extrafascial technique by means of clamps and suture ligation^(9)^. The technical aspects of both types of hysterectomy were discussed with each patient, and the appropriate hysterectomy type was selected through mutual discussion.

### Questionnaires

Sexual dysfunction and quality of sexual life, psychological health status, and self-esteem were assessed preoperatively and 6 months postoperatively using standardized validated questionnaires: the Arizona Sexual Experiences Scale (ASEX), the Symptom Checklist-90-Revised (SCL-90-R), and the Rosenberg Self-Esteem Scale (RSES).

### Arizona Sexual Experiences Scale

The patients’quality of sexual life was measured using the ASEX scale. The ASEX is a five-item scale with six different levels of answers that measure sexual function. The questionnaire enquires about sex drive, arousal, vaginal lubrication, ability to reach orgasm, and satisfaction with orgasm. The female and male versions of the ASEX differ on the sex-specific question 3, which addresses erection/lubrication. A total ASEX score of ≥19, any one item with an individual score of 5 or 6, and three or more items with individual scores of 4 have all been found highly correlated with physician-diagnosed sexual dysfunction^(10)^. Patients whose partners had sexual dysfunction were eliminated from the study. We used the Turkish version of the inventory, which has been proven valid and reliable by a recent study (Cronbach’s alpha=0.91)^(11)^.

### Symptom Checklist-90-Revised Scale

SCL-90-R was used to evaluate the psychological health status of the patients. The SCL-90-R includes items about psychosomatic symptoms of the patient and covers nine scales:

1. Somatization;

2. Obsessive-compulsive behavior;

3. Interpersonal sensitivity;

4. Depression;

5. Anxiety;

6. Hostility;

7. Phobic anxiety;

8. Paranoid ideation;

9. Psychoticism^(12,13)^.

Stressful personality, depression, anxiety, and somatization are generally measured using five-point scales (sores between 0-4)^(14)^. Increasing scores generally indicate that the patient is anxious about the symptoms and signs. We used the Turkish version of the inventory, which has been proven valid and reliable by a recent study (Cronbach’s alpha=0.83)^(15)^.

### Rosenberg Self-Esteem Scale

RSES consists of 10 items for assessing levels of self-esteem. The items are answered on a four-point scale using anchors of strongly agree (0) and strongly disagree. Its reliability and validity in Turkish were confirmed in 1986 by Çuhadaroğlu^(16)^ and the first 10 items of the test, which assess self-esteem, were used. The subjects achieve scores between 0 and 6 according to the self-assessment system of the scale. A score of 0-1 is considered high self-esteem, a score of 2-4 is considered moderate self-esteem, and a score of 5-6 is considered low self-esteem. A high score indicates low self-esteem, whereas a low score indicates high self-esteem.

### Statistical Analysis

Statistical analysis was performed using SPSS version 21.0 for Windows. Values are expressed as mean ± standard deviation. The Kolmogorov-Smirnov test was performed to assess the distribution of data. A comparison of two groups was performed using Student’s t-test or Mann-Whitney U test for continuous variables, and the chi-square test for categorical variables. Comparisons of preoperative and postoperative scores were performed using the paired-samples t-test and Wilcoxon test. A p value of <0.05 was considered to indicate statistical significance.

## RESULTS

**Figure 1:**
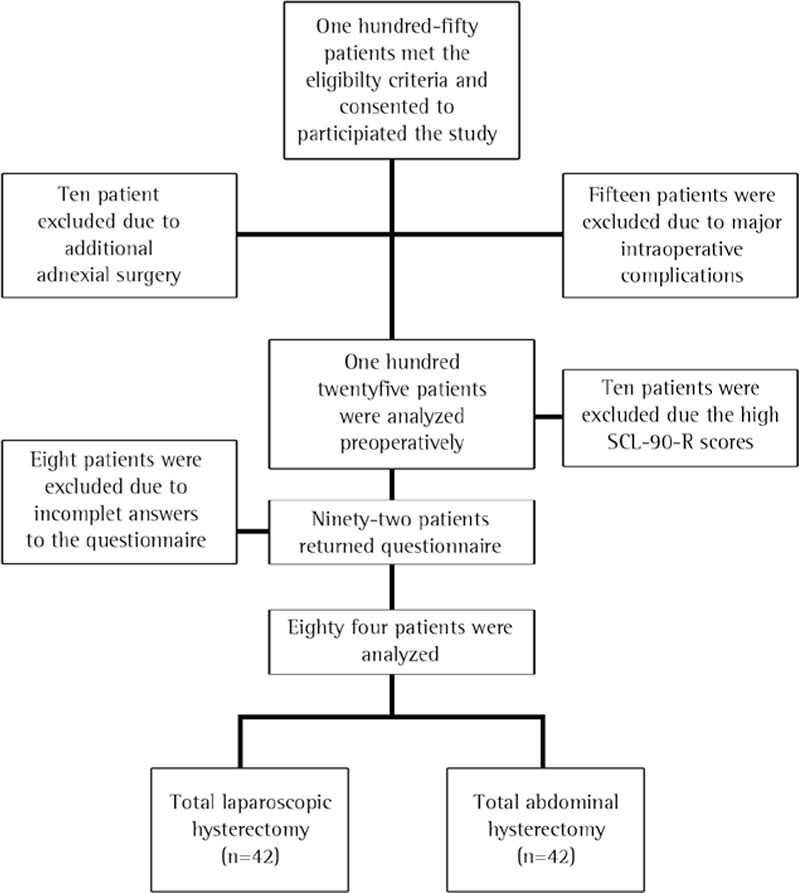
Flow chart of study design *SCL-90-R: Symptom Checklist-90-Revised*

The patient flow chart is listed in detail in Figure 1. Out of 150 eligible patients, 84 completed the study. The demographic and clinical data of the two groups are summarized in Table 1.

There were no statistically significant differences in the pre-and postoperative SCL-90-R scores between the groups (p>0.05) (Table 2).

Both groups exhibited a statistically significant decrease in the postoperative RSES scores compared with the preoperative scores (p<0.05). However, there were no statistically significant differences between the groups (p>0.05) (Table 3). Evaluations of sexual function are summarized in Table 4. No differences were observed between the two groups preoperatively. Postoperative decreases in ASEX scores (improvement in sexual function) were observed in both groups. Improvement in sex drive score and psychological arousal scores were better in the laparoscopic hysterectomy group compared with the abdominal group (p<0.01).

## DISCUSSION

In the present study, decreased ASEX scores were observed 6 months postoperatively in both groups. This was more remarkable in the laparoscopy group compared with the laparotomy group. Both groups showed improvement in RSES scores; however, no significant difference was observed. Regarding the psychiatric evaluation, there were no significant differences between the groups preoperatively and 6 months postoperatively. The effects of hysterectomy on women’s sexuality are controversial, and sexual function in the post-hysterectomy period is a complicated and uncertain issue by means of its results^(17,18,19,20,21)^. Hysterectomy may increase the quality of life in patients who did not respond to conservative therapy by relieving symptoms^(22)^. Nevertheless, patients preparing for hysterectomy may experience fear and anxiety of sexual function loss^(23)^. Hysterectomy is a loss because the uterus is an organ to which women are connected psychologically. Psychosexual problems after hysterectomy are usually related to marital issues and poor body image, which are exacerbated with hysterectomy. Age, biologic and psychological factors, relationships and social and cultural circumstances affect a woman’s sexuality^(2,24)^. Therefore, women’s sexuality needs to be assessed through many independent factors. On the other hand, preoperative sexual function and psychological status are also important determinants of sexual dysfunction postoperatively. In our study, essential factors affecting sexuality such as self-respect and psychological status were evaluated. We observed no statistically significant changes in psychiatric scores in either group; however, amelioration of self-respect was observed postoperatively, and in our opinion, this may be a substantial factor affecting sexual improvement. In order to ensure a homogenous study population, we excluded patients with high preoperative psychiatric scores. Thus, we aimed to assess the effect of the operation on sexual life and also on the patient’s psychology. Following hysterectomy, elimination of pain, discomfort, resistant menometrorrhagia associated with present disease, and risk of cancer and unwanted pregnancy may result in higher frequency of orgasms and a more satisfying sexual life^(25)^.

Farrell and Kieser^(26)^ claimed in their study that improvement in quality of life and no negative effects on sexuality were observed. The most important factor they emphasized was that there were many factors influencing sexual life and that these should be assessed in unity.

Ayoubi et al.^(2)^ compared three types of hysterectomy (vaginal, abdominal, and laparoscopic) and found no differences in the effects on orgasm, frequency of sexual intercourse and sexual desire between groups; however, poorer body image was observed less in the TLH group compared with the TAH group. Another important finding of this study was that adverse psychological effects were observed less in the laparoscopy group. The absence of abdominal scars in the laparoscopy group and less postoperative pain may explain these findings.

A study that compared 5 different hysterectomy procedures conducted by Lermann et al.^(27)^ reported that women in the laparoscopic supracervical hysterectomy (LASH) and TLH groups had more favorable results. However, the difference between TLH and LASH groups was not statistically significant. The study of Lermann et al.^(27)^ was a long-term and broad study but the retrospective study design and lack of sexual evaluation before surgery were important limitations of the study. Although statistical significant differences between the groups were not encountered, the conclusion of less invasive methods had more favorableresults was compatible with our results.

Gutl et al.^(28)^ compared vaginal hysterectomy (VH) and TAH groups. The authors assessed patients 3 months and 2 years postoperatively and observed improvement on sexual function in both groups; more pain and poor self-image were observed in the TAH group, which may be associated with abdominal scar appearance. In addition, the recovery period was longer compared with the VH group. Hehenkamp et al.^(29)^ randomly assigned patients to undergo uterine artery embolization and hysterectomy for the treatment uterine fibroids, then assessed sexual activity and body image scales in both groups. Improvement was more apparent in the uterine artery embolization group. Less invasive methods of surgery appear to have a positive impact on quality of life and patient comfort. This favorable change in self-body image and quality of life also has indirect positive repercussions on sexual life. Both TLH and TAH groups showed decreased postoperative ASEX scores. In comparison with abdominal operations, sexual drive scores and arousal scores decreased more in the TLH group, which indicated improvement in sexual function. TLH appears to have advantages for women who require total hysterectomy for benign indications, particularly with regard to sexual functions. The main strengths of our study were the prospective observational design and the patients were chosen homogeneously. However, the small number of the study group and short follow-up period (6 months postoperatively) were the main limitations of the study.

## CONCLUSION

In conclusion, laparoscopic surgery should be performed on suitable patients considering that it is less invasive, has a shorter recovery period, and has positive effects on sexual function and quality of life. We think that further research with a prospective long-term follow-up design is necessary to identify a surgical option associated with maximum preservation of sexual function during hysterectomy procedures.

## Figures and Tables

**Table 1:**
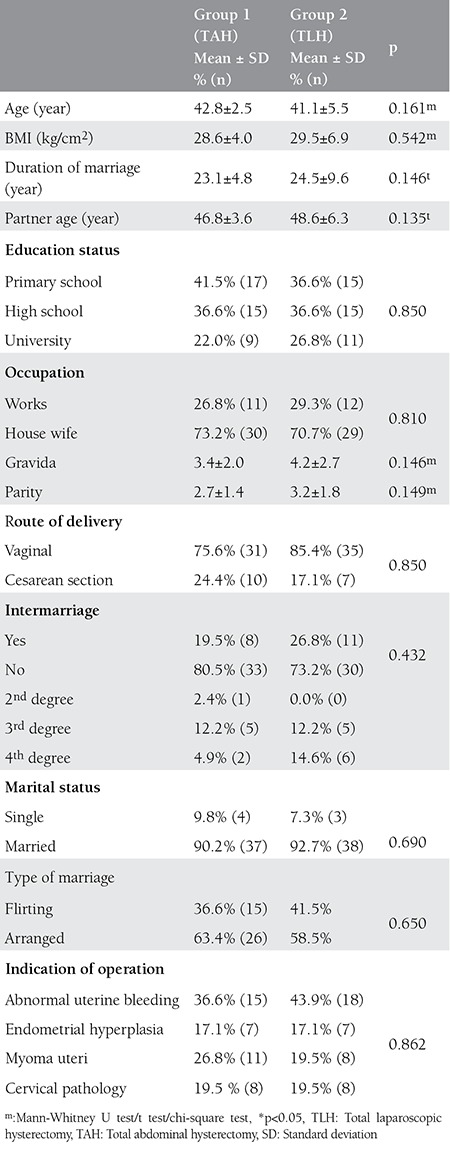
Patients’ sociodemographic variables and medical characteristics prior to surgery

**Table 2:**
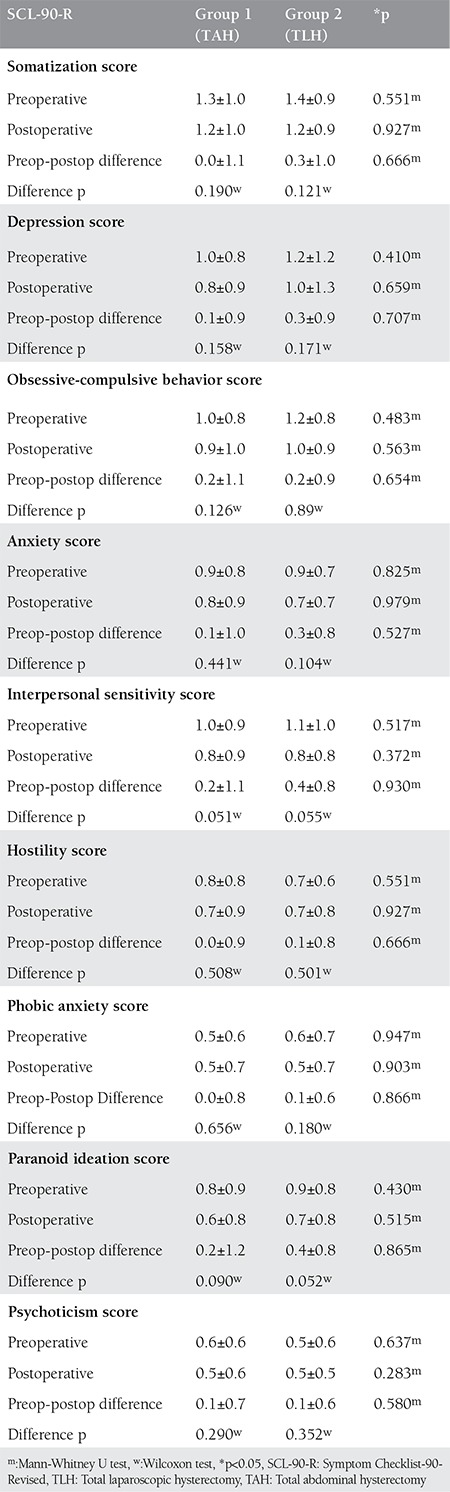
Comparison of Symptom Checklist-90-Revised preoperative and postoperative 6^th^ month

**Table 3:**
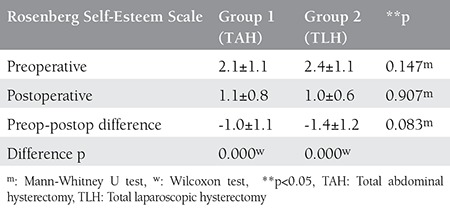
Comparison of Rosenberg Self-Esteem Scale preoperative and postoperative 6^th^ month

**Table 4:**
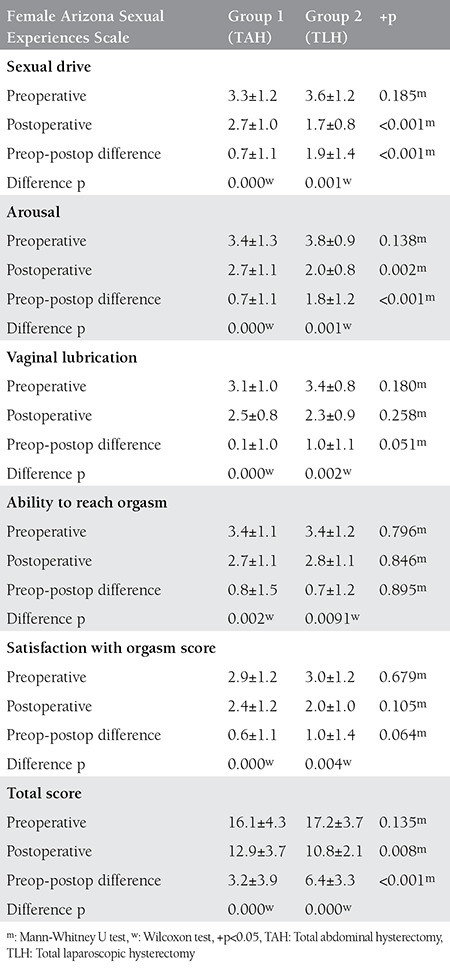
Comparison of Arizona Sexual Experiences Scale score preoperative and postoperative
